# Study of class 1 integrons in multidrug-resistant uropathogenic *Escherichia coli* isolated from different hospitals in Karachi 

**DOI:** 10.22038/IJBMS.2018.28807.6966

**Published:** 2018-10

**Authors:** Fouzia Zeeshan Khan, Tehseen Nawaz, Zulfiqar Ali Mirani, Saeed Khan, Yasir Raza, Shahana Urooj Kazmi

**Affiliations:** 1 Dow University of Health Sciences, Karachi, Pakistan; 2 Barrett Hodgson University, Karachi, Pakistan; 3 Pakistan Council of Scientific & Industrial Research Laboratories Complex, Karachi, Pakistan; 4 Department of Microbiology, University of Karachi, Karachi, Pakistan; 5 Dada Bhoy Institute of Higher Education Commission, Karachi, Pakistan

**Keywords:** Class 1 integrons, ESBLs, MDR E. *coli*, Multidrug resistance, Uropathogenic E. *coli*

## Abstract

**Objective(s):**

*Escherichia coli *is the key pathogen in the family producing ESBL (extended spectrum β-lactamase) and associated with community-acquired infections. Therefore, this study was planned to determine the antibiotic susceptibility pattern of uropathogenic *E. coli*, prevalence of the ESBL gene group and class 1 integrons.

**Materials and Methods:**

Clinical isolates of uropathogenic *E. coli* were isolated from different hospitals of Karachi. Antibiotic susceptibility test was performed by Kirby-Bauer Methods. Presence of β– lactamases genes (CTX, TEM, and SHV) and integron 1 were identified by polymerase chain reaction (PCR).

**Results:**

Out of 500, 105 isolates were identified as multi-drug resistant (MDR) uropathogenic* E. coli*. The subject MDR isolates showed the highest resistance to aztreonam, amoxil/ clavulanic acid, ampicillin, cotrimoxazole, ceftriaxone, cefipime, and cefuroxime. Genetic analysis showed that the majority of the MDR *E. coli *carry CTX M1 (57.1%) followed by TEM (33.3%) and SHV (9.5%). Moreover, 79% of MDR* E. coli *harbored class 1 integrons, whereas all three conserved genes for class 1 integrons were present in 58% of MDR *E. coli*.

**Conclusion:**

This study is helpful to provide information regarding the antibiotic susceptibility pattern, distribution ESBLs and class 1 integrons among uropathogenic *E. coli*.

## Introduction

*Enterobacteriaceae* is a diverse group of Gram-negative bacteria known for their versatile pathogenicity (1, 2). *Escherichia* *coli* is one of the major pathogens in this family and associated with antibiotic resistance through ESBL (extended spectrum β-lactamase) production (3, 4). The ESBLs are a group of enzymes that hydrolyze oxyimino cephalosporins and monobactams except cephamycin. The genes for β-lactamases and ESBLs are carried by plasmids as well as chromosomes (5). Point mutation of amino acid in parent β-lactamases (TEM-1, TEM-2, and SHV) led to the formation of ESBL. In late 1900’s and early 2000’s, a new ESBL, CTX-M enzymes were discovered from *E. coli,* which is associated with community-acquired urinary tract infections (6). The CTX-M β-lactamases play a major role in resistance against third-generation cephalosporin especially cefotaxime (7). Commensal *E. coli *strains are very active in the interchange of genetic material with different pathogenic bacteria e.g., *Salmonella*, *Shigella*, *Vibrio* and *Yersinia* (8, 9). The dissemination of various genes is responsible for antibiotic resistance and also related to genetic structures called integrons (10). In recent years, integrons play a major role in the horizontal transmission of antibiotic resistance genes in bacteria. Integron has a specific site, *attI1*, at which gene cassettes can be incorporated by site-specific recombination, encoded by an integrase gene (intI1) (11). Class 1 integrons are responsible for multidrug resistance as observed in *E. coli* (12). 

It is important to understand the molecular mechanism of resistance, which may help to develop new techniques for preventing the spread of resistance determinants among the pathogens. The aim of this study is to check the antibiotic susceptibility pattern of uropathogenic *E. coli, *prevalence of the ESBL gene group and class 1 integrons.

## Materials and Methods

***Isolation and biochemical identification of bacterial isolates***

A total of 500 *E. coli* isolates were collected from different hospitals of Karachi and cultured on cysteine lactose electrolyte deficient media (CLED) (Oxoid). The subject isolates were further confirmed as *E. coli* on the basis of colony morphology, gram staining reaction, motility and biochemical tests such as pigmentation and fermentation of glucose, mannitol, Voges–Proskauer(VP) and nitrate test, oxidase production, sulfide reduction, indole production, and molecular characterization. *E. coli* ATCC 25922 was used as a control in the study.

**Table 1:** List of target genes and primers used for molecular characterizations of MDR uropathogenic *Escherichia coli*

Gene	Primer	Sequence	
16SrRNA	341F	TAC GGG AGG CAG CAG	
	518R	ATT ACC GCG GCT GCT GG	
UID A gene	EC-F	ATCACCGTGGTGACGCATGTCGC	
	EC-R	CACCACGATGCCATGTTCATCTGC	
CTX	F	CGTCACGCTGTTGTTAGGAA	
	R	ACGGCTTTCTGCCTTAGGTT	
TEM	F	TCGGGGAAATGTGCG	
	R	TGCTTAATCAGTGAGGCACC	
SHV	F	GCCGGGTTATTCTTATTTGTCGC	
	R	ATGCCGCCGCCAGTCA	
intI1	F	GGTTCGAATGTCGTAACCGC	
	R	ACG CCCTTGAGCGGAAGTATC	
qacE∆1	F	GAGGGCTTTACTAAGC TTGC	
	R	ATACCTACAAAGCCCCACGC	
sul1	F	ATCAGACGTCGTGGATGTCG	
	R	CGAAGAACCGCACAATCTCG	

**Figure 1:**
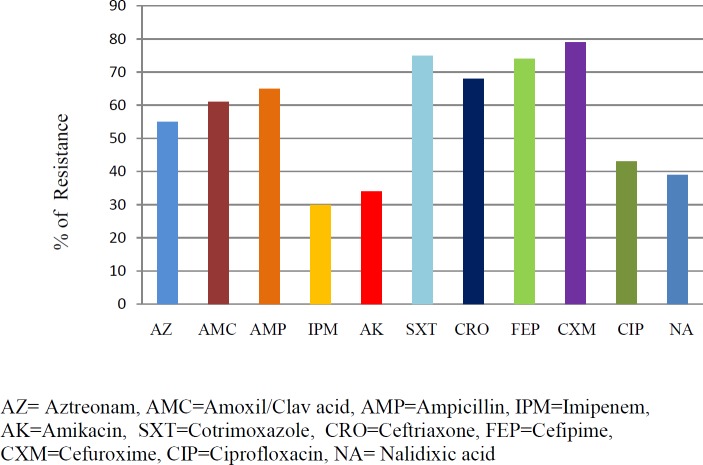
Antibiotic susceptibility pattern of MDR uropathogenic *Escherichia coli*

***Antimicrobial susceptibility pattern***

Antimicrobial susceptibility of uropathogenic *E. coli* was performed on Mueller-Hinton agar (MHA) by Kirby-Bauer disc diffusion method according to the guidelines of the Clinical & Laboratory Standards Institute (CLSI) 2012 (13). 

**Figure 2:**
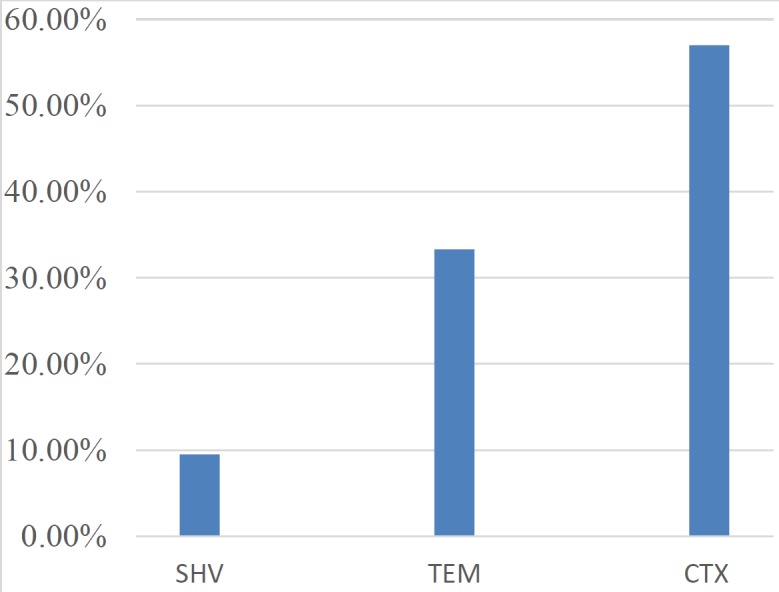
Distribution of ESBL s genes of MDR *Escherichia coli*

**Table 2:** Frequency of class 1 integron genes isolated from MDR* Escherichia coli*

qacEΔ1+int1+sul1 58%	58%
int1+sul1 10%	10%
sul1+ qacEΔ1 5%	5%
qacEΔ1 6%	6%
None 21%	21%

Antibiotics such as ampicillin (AMP), aztreonam (AZ), amoxicillin/clavulanic acid (AMC), imipenem (IPM), amikacin (AK), ceftriaxone (CRO), cefepime (FEP), cefuroxime (CXM), ciprofloxacin (CIP), nalidixic acid (NA), piperacillin-tazobactam (TZP), and cotrimoxazole (SXT) were tested.

***Molecular study***

Strains identified as MDR uropathogenic *E. coli *were cultured on Muller-Hinton agar (MHA) and total genomic deoxyribonucleic acid (DNA) was extracted by the SDS-PK method (14). PCR was conducted by targeting the 16srRNA gene with primers 341F and 518R (15). The uropathogenic *E. coli* were confirmed by amplification of *uid A* gene encoding *β*-glucuronidase using primers ECF and ECR (16). All 105 MDR *E. coli* were evaluated for the presence of ESBL gene group TEM, SHV, and CTX M 1 by using specific primers and conditions (17-19). Integrons were detected by using multiplex PCR, targeting three conserved sequences of class 1 integrons (intI1, qacE∆1, and sul1) as discussed by Ebner *et al*. (20). 

***Statistical analysis***

Data analysis was accomplished by using the Statistical Package for Social Science (SPSS ver. 17.0) for frequencies.

## Results

Out of 500 uropathogenic *E. coli* isolates, 105 isolates were identified as MDR *E. coli* on the basis of antimicrobial sensitivity pattern which exhibited that >50% of the subject isolates were resistant to aztreonam, amoxil/clav acid, ampicillin, cotrimoxazole, ceftriaxone, cefipime, and cefuroxime. About 40% of the targeted isolates exhibited resistance to imipenem, amikacin, and nalidixic acid (Table 1). These isolates were further confirmed as MDR *E. coli* by the amplification of 16srRNA and UID A genes. Interestingly, all 105 MDR *E. coli* isolates were found to carry ESBL genes. Comparative analysis showed that the majority of the isolates (57.1%) carry CTX M1 type ESBL, followed by TEM (33.3%) and SHV (9.5%) (Figure 2). Presence of Class 1 integron is confirmed by the amplification of *intI1 *in 79% of MDR *E. coli.* All three conserved genes *intI1*, *qacE*Δ*1,* and *sul1* were found in 58% of MDR *E. coli*. *qacE*Δ*1* and *sul1* were found to be present in 5% of isolates, whereas, *intI*, *sul1,* and q*acE*Δ*1* were present in 10%, 10%, and 6% of isolates, respectively. The absence of class 1 integrons was noticed in 21% of isolates as shown in Table 2. 

## Discussion

Extended-spectrum β-lactamases are known to constitute an important mechanism of resistance against β-lactam classes of antibiotics in *Enterobacteriaceae* (21). These enzymes are coded by genes located on the bacterial chromosome as well as on plasmids and are interchangeable in between bacteria (22). Urinary tract infections are the most commonly occurring infectious diseases caused by *Enterobacteriaceae* especially *E. coli* (23). It is found that the incidence of ESBL-producing *E. coli* in high-risk parts of the hospital settings, such as ICUs and CCU, has prominently increased (24). In our study, UTIs were identified from out-patients, which shows a significant spread of ESBLs in the community; although these outpatients’ infections are not completely community-based and may be associated with hospital follow up checkups. MDR *E. coli* is exhibiting an increasing trend of resistance, more than 50% resistance is observed in antibiotics, including aztreonam, ceftriaxone, cefipime, cefuroxime, and cotrimoxazole, whereas, less than 50% resistance is found against imipenem and quinolones. Findings of the present study correlated with the results of a recent study in Pakistan (25). Furthermore, a study conducted by the European Antibiotic Resistance Surveillance System demonstrated an ascending pattern of resistance to third-generation cephalosporin reported from 31 countries in 800 laboratories (26). Antibiotic treatment for infections related to ESBL producing *E. coli* is severely affected with cross-resistance to other antibiotics such as quinolones. The present study also demonstrated co-resistance of cephalosporins and quinolones groups.

 Bidel *et al*. confirmed our findings and suggested that quinolone resistance could be the result of indiscriminate use of cephalosporin at both hospital settings and community levels (27). In this perspective, carbapenems are pretty good alternative options for therapies but our study reported resistance against imipenem. This was also endorsed by the results of a study that discussed the genetic characterization of carbapenem-resistant *E. coli *(28). In addition to plasmids and chromosomes, the genes regulating ESBLs are also carried on integrons, which are mainly responsible for dissemination of multidrug resistance (29). Class A ESBLs including CTX-M, SHV, and TEM are responsible for dissemination of β-lactam resistance among members of *Enterobacteriaceae,* and are located on plasmids as well as on integrons (30). Interestingly, more than 300 different variants of ESBL including TEM and SHV have been documented, however, CTX-M is the major enzyme carried by the majority of *Enterobacteriaceae* members (31). Comparative analysis during the present study indicates that the majority of MDR *E. coli* harbor CTX M1 followed by TEM and SHV. Earlier studies showed a high prevalence of CTX M1 gene, which is increasingly linked with community-acquired infections and high mobilization of the encoding genes (32-34). Generally, CTX-M-β-lactamases were found to be effective against cefotaxime, but nowadays more than 60% of CTX M variants are resistant to both ceftazidime and cefotaxime. They also exhibit cross-resistance against fluoroquinolones, aminoglycosides, and cotrimoxazole (35-37). 

The isolates of *E. coli* harboring CTX M were found to be more pathogenic and responsible for severe infections (38). The co-occurrence of CTX, SHV, and TEM genes in our report could be responsible for the resistance against carbapenem, supported by a study conducted in India (39). Additionally, the present study indicates that 79% of MDR* E. coli* harbored all three conserved genes of integron class 1. This is a rare phenomenon because other studies reported 16–59% prevalence of integron class 1 in MDR* E. coli. *The difference may be due to the selection bias of MDR *E. coli *resulting in a geographical variation in the distribution of integrons (40, 41). Furthermore, a higher number possessed *intI1* and *sul1* as compared to the occurrence of a combination of *qacE*Δ*1*, *sul1,* and *qacE*Δ*1.* This is the major factor behind cross-resistance as well as multi-drug resistance in isolates of MDR *E. coli* used in the present study. The absence of *qacE*Δ*1* and *sul1* along with *intI1 *suggests that either Class I integrons have lost the sul1 and *qacE*Δ*1* gene regions or that these genes are carried on another genetic context in these strains, as supported by other studies (42, 43).

## Conclusion

Uropathogenic ESBL-producing *E. coli* is an emerging health issue spreading worldwide. This information would be helpful in understanding the epidemiology of resistance as well as the application of recommendations for the proper controlling of antimicrobials usage and infection control measures. Therefore, active surveillance and antimicrobial stewardship are highly recommended.
